# A phase II study of gemcitabine, erlotinib and S-1 in patients with advanced pancreatic cancer

**DOI:** 10.7150/jca.50514

**Published:** 2021-01-01

**Authors:** Boram Han, Bum Jun Kim, Hyeong Su Kim, Dae Ro Choi, Byoung Yong Shim, Kyung Hee Lee, Jin Won Kim, Jung Han Kim, Hunho Song, Jong Hyeok Kim, Choong Kee Park, Jung Woo Lee, Min-Jeong Kim, Dae Young Zang

**Affiliations:** 1 Department of Internal Medicine, Hallym University Medical Center, Hallym University College of Medicine, Anyang-si, Gyeongigi-do, Korea.; 2 Department of Internal Medicine, St. Vincent's Hospital, College of Medicine, The Catholic University of Korea, Suwon, Korea.; 3 Department of Internal Medicine, Yeungnam University Medical Center, Yeungnam University College of Medicine, Daegu, Korea.; 4 Department of Internal Medicine, Seoul National University Bundang Hospital, Seoul National University College of Medicine, Seongnam-si, Gyeonggi-do, Korea.; 5 Department of General Surgery, Hallym University Medical Center, Hallym University College of Medicine, Anyang-si, Gyeongigi-do, Korea.; 6 Departement of Radiology, Hallym University Medical Center, Hallym University College of Medicine, Anyang-si, Gyeongigi-do, Korea.

**Keywords:** pancreatic neoplasms, gemcitabine, erlotinib, S-1, phase II study

## Abstract

**Background:** We evaluated the efficacy and safety of gemcitabine in combination with erlotinib and S-1 for the treatment of advanced pancreatic cancer.

**Methods:** Chemotherapy-naïve patients with pathologically-proven locally advanced, recurrent, or metastatic pancreatic adenocarcinoma were assessed for eligibility. Gemcitabine was administered at 1,000 mg/m^2^ intravenously on days 1 and 8, erlotinib was administered at 100 mg/day on days 1-21, and S-1 was administered at 60 mg/m^2^ on days 1-14 every 21 days and continued to a maximum of 8 cycles of treatment. Dose escalation of S-1 to 80 mg/m^2^ was permitted from the second cycle for pre-defined tolerable patients.

**Results:** Thirty-seven patients (median age 61.5 years) were enrolled. A total of 140 cycles of chemotherapy were administered (median of 3.8; range 1-8 cycles). Toxicities were evaluated in 36 patients, and the responses were evaluated in 32 patients. Major grade 3/4 toxicities included neutropenia (25%), febrile neutropenia (2.8%), fatigue (22.2%), infection (8.3%), vomiting (5.6%), and mucositis (5.6%). The confirmed overall response rate was 12.5% [95% confidence interval (CI), 5.1-28.9%] and the disease control rate was 71.9% (95% CI, 56.8-86.3%). The median progression-free survival and overall survival were 3.7 months (95% CI, 2.8-4.6 months) and 6.7 months (95% CI, 3.4-9.9 months), respectively.

**Conclusion:** The combination of gemcitabine, erlotinib, and S-1 provided an acceptable toxicity profile and modest clinical benefits in patients with advanced pancreatic cancer.

## Introduction

Pancreatic ductal adenocarcinoma (PDAC) is an intractable disease and is the 7^th^ leading cause of global cancer deaths in industrialized countries 1. Because more than 80% of PDAC is locally advanced unresectable or metastatic at the time of diagnosis 2, the prognosis of PDAC patients is dismal with a 5-year relative survival rate of 11.4% 3. Although various types of targeted agents and immunotherapeutic agents are actively used in other cancers, cytotoxic chemotherapy remains the mainstream treatment for unresectable PDAC.

Following the approval of gemcitabine by the US Food and Drug Administration in 1997 4, gemcitabine-based chemotherapy was considered the standard of care for patients with advanced PDAC for a decade. In the era of gemcitabine, various attempts were made simultaneously to find an optimal drug combination that could function synergistically with gemcitabine. A number of drugs, including cytotoxic agents 5-10 and targeted agents 11, 12, in combination with gemcitabine, were tested in large randomized clinical trials, but they failed to improve the efficacy.

Among the various drugs investigated, erlotinib, a small-molecule inhibitor of epidermal growth factor receptor, improved the efficacy of gemcitabine in a randomized phase III trial 13. In this study, erlotinib in combination with gemcitabine showed a small but statistically significant improvement in overall survival when compared to gemcitabine monotherapy (6.2 months vs. 5.9 months, *p*=0.038). S-1, an oral fluoropyrimidine derivative, was also studied as a combination partner of gemcitabine and has consistently shown promising results in terms of efficacy and safety in a series of phase II studies 14-18.

At the time this study was proposed and designed, gemcitabine plus erlotinib combination chemotherapy was approved as front-line chemotherapy for unresectable PDAC and was widely used globally. However, since the benefit of gemcitabine plus erlotinib in survival prolongation was too small, there has been a continuing need for new drugs or combination regimens for patients with PDAC. In this background, combination therapy with gemcitabine, erlotinib, and S-1 (GTS regimen) has been proposed as a novel front-line treatment for unresectable PDAC, and this study was conducted to demonstrate the efficacy and safety of this regimen.

With two combination regimens of FOLFIRINOX (a combination of oxaliplatin, folinic acid, irinotecan, and fluorouracil [5-FU]) 19 and albumin-bound paclitaxel/gemcitabine 20 currently accepted as front-line treatments and actively used in fit patients, the clinician's interest and possible range of application for our combination regimen will be limited. However, since GTS combination therapy has never been investigated in PDAC, it would be valuable to report and share efficacy and safety data.

## Methods

### Patient eligibility

Patients were eligible for this study if they fulfilled all of the following criteria: (1) pathologically confirmed unresectable locally advanced, recurrent, or metastatic adenocarcinoma of the pancreas; (2) measurable disease, as defined using version 1.1 of the Response Evaluation Criteria In Solid Tumors (RECIST); (3) age ≥18 years; (4) Eastern Cooperative Oncology Group (ECOG) performance status of 0-1; (5) prior adjuvant chemotherapy without gemcitabine, erlotinib, or S-1 that had been completed >4 weeks before enrollment; (6) more than 4 weeks since completion of prior radiotherapy (measurable lesions are outside the radiation field); (7) adequate hematological, renal, and hepatic functions, as defined using an absolute neutrophil count of ≥1.5 × 10^9^/L, a platelet count of ≥100 × 10^9^/L, serum creatinine levels of ≤1.5 × upper limit of normal or creatinine clearance ≥50 mL/min, serum bilirubin ≤2× UNL, aspartate aminotransferase and alanine aminotransferase levels of ≤2.5×; and, (8) willingness to provide informed consent to participate in this study.

Patients were excluded based on the following criteria: (1) a history of treatment with gemcitabine, erlotinib, or S-1 as adjuvant chemotherapy; (2) contraindication for any drug contained in the chemotherapy regimen; (3) central nervous system metastasis; (4) serious GI bleeding or obvious bowel obstruction; (5) other previous or concurrent malignancies within the last 5 years, with the exception of cured basal cell carcinoma of the skin or carcinoma *in situ* of the uterine cervix; (6) pregnant or lactating female patients; (7) sexually active and the partner is unwilling to practice contraception during the study; and (8) other clinically significant comorbid conditions, such as an active infection or severe cardiopulmonary dysfunction.

### Treatment and study design

The treatment consisted of intravenous administration of gemcitabine at 1,000 mg/m^2^ on days 1 and 8 every 3 weeks, continuously orally administered erlotinib at 100 mg/day, and orally administered S-1 at 30 mg/m^2^ twice daily on days 1-14 of each cycle. Patients with a body surface area of <1.25 m^2^ received 80 mg of S-1 daily, those with a body surface area of 1.25-1.5 m^2^ received 100 mg of S-1 daily, and those with a body surface area of ≥1.5 m^2^ received 120 mg of S-1 daily. Treatment was delivered as a 3-week cycle and repeated up to a maximum of 8 cycles of chemotherapy, or until disease progression, unacceptable toxicity, or the patient's refusal.

This trial was a prospective, single-arm phase II study evaluating combination chemotherapy with gemcitabine, erlotinib, and S-1 in previously untreated patients with unresectable locally advanced or metastatic pancreatic cancer. The primary endpoint was the confirmed objective response rate (ORR), and the secondary endpoints were median progression-free survival (PFS), median overall survival (OS), disease control rate (DCR), and toxicity profiles. The investigation was performed in accordance with the Declaration of Helsinki, and the protocol was approved by the institutional review boards of Hallym University Medical Center, Anyang-si, South Korea, and Asan Medical Center, Seoul, South Korea (protocol number: HMC-HO-GI-1201).

### Dose modifications and dose intensity

Dose modifications were performed according to the study protocol. The next treatment cycle was initiated only when the neutrophil count was 1.5 × 10^9^/L or greater and the platelet count was 100 × 10^9^/L or greater. Treatment was delayed in the event of grade 3/4 nonhematologic toxicities until the toxicities were resolved to grade 1 or lower. The doses of gemcitabine and S-1 were reduced by 25% of the initial doses for related grade 3/4 neutropenia, grade 3 febrile neutropenia, grade 3 thrombocytopenia, or for the second occurrence of the same grade 2 neutropenia and thrombocytopenia. The doses of gemcitabine were reduced by 50% of the initial doses for grade 4 thrombocytopenia, or for the second occurrence of the grade 3/4 neutropenia, grade 3 febrile neutropenia, grade 3 thrombocytopenia, or for the third occurrence of grade 3 neutropenia and thrombocytopenia. In the case of the second occurrence of grade 2 thrombocytopenia, grade 3/4 neutropenia, grade 3 febrile neutropenia, or the third occurrence of grade 2 neutropenia, erlotinib was omitted until recovery and then re-challenged. Treatment was discontinued if, despite the dose reduction, the same toxicity occurred for a fourth time at grade 2, a third time at grade 3, or a second time at grade 4 or any occurrence of life-threatening sepsis during treatment. In addition, if the toxicity had not improved to grade 0 or 1 after 3 weeks, the patient was withdrawn from the study. The dose reduction was maintained in subsequent cycles.

To evaluate a function of the drug and the frequency of administration, we calculated the relative dose intensity (RDI), which is expressed as the ratio of the administered amount of dose per time unit (mg/m^2^/week) to that of the originally planned dose.

### Toxicity and response evaluation

A physical examination with vital signs, complete blood cell counts with differentials, and blood chemistry tests were performed before every administration of gemcitabine in each subsequent cycle. Toxicity was evaluated and graded according to version 4.0 of the Common Terminology Criteria for Adverse Events of the National Cancer Institute. All of the patients who received at least one dose of treatment were included in the toxicity assessment. For the toxicity analysis, the data indicating the worst toxicity for each patient from all of the chemotherapy cycles were used. The proportion of patients who experienced adverse events was calculated by dividing the number of patients who experienced adverse events during the treatment period by the number of patients evaluable for safety analysis. Response to treatment according to RECIST version 1.1 was evaluated every 2 cycles. Patients with CR or PR required a confirmatory disease assessment at least 4 weeks later. PFS was defined as the interval from the date of treatment initiation to the first date of documented disease progression or death due to any cause. OS was defined as the interval from the date of treatment initiation to the date of death.

### Statistical analysis

According to Simon's optimal two-stage design, 25 patients were required for enrollment to test the null hypothesis that the true ORR is 10% versus the alternative hypothesis that the true ORR is at least 30%, at a significance level of *p*<0.05 with a power of 80%. If two or more responses were observed among 15 patients in the first stage, the study was continued with 10 additional patients included. As the drop-out rate was assumed to be 10%, the number of patients necessary for recruitment into the study was calculated to be 28.

Descriptive statistics were used to summarize the patients' characteristics, tumor responses, and safety events. The Kaplan-Meier method was used to estimate the median PFS and OS. All enrolled patients were included in an intent-to-treat analysis.

## Results

### Patient characteristics

From October 2012 to May 2016, 37 patients who met the inclusion criteria were enrolled in this study. We exceeded the planned number of patients because several unexpected dropouts occurred early in the study and we allowed simultaneous registration of excess patients before the end of the study from multiple institutions. The reasons for dropout are explained below. The demographic and pathologic characteristics of the patients are described in **Table 1.** The median age was 61.5 years (range 35-88 years). Sixteen patients (43.2%) were male, and the majority of patients (73.0%) had an ECOG PS of 1. Twenty-six patients (70.3%) had metastatic disease, eight patients (21.6%) had recurrent pancreatic cancer after curative surgery, and three patients (8.1%) had locally advanced disease at the time of screening. The most common metastatic sites were distant lymph nodes (43.2%), the liver (43.2%), the lung (29.7%), and the peritoneum (27.0%).

### Treatment administration

In total, 140 treatment cycles were administered to 37 patients, with a median of 3.8 cycles (range 1-10 cycles) per patient. Five patients did not complete the first cycle of chemotherapy: two patients died (one patient died of cerebral infarction and one patient died of hepatic tumor rupture), two patients withdrew their informed consent, and one patient was lost to follow-up. Seven patients (18.9%) completed eight or more cycles of chemotherapy. Eleven patients (29.7%) required dose reductions or delays. The mean relative dose intensities (ratio of the dose received to the dose planned) of gemcitabine, S-1 for all of the cycles administered were 0.87 [95% confidence interval (CI) 0.81-0.93], and 0.92 (95% CI 0.87-0.96), respectively (**Table 2**).

### Efficacy

Of the 37 patients, 32 were eligible for response evaluation. Five patients were not available for response evaluation: the detailed reasons for 5 patients who did not complete the first cycle are described in the '*Treatment administration*' section. The tumor responses are summarized in **Table 3.** There were 4 partial responses, 19 cases of stable disease, and 9 cases of disease progression. All partial responses are confirmed in the following CT scan. The confirmed ORR was 12.5% (95% CI 5.1-28.9%) and the disease control rate was 71.9% (95% CI 56.8-86.3%). The median time to response was 1.4 months (95% CI 1.3-1.5 months) and the median duration of response was 7.4 months (95% CI 3.8-11.0 months).

**Figure 1:**
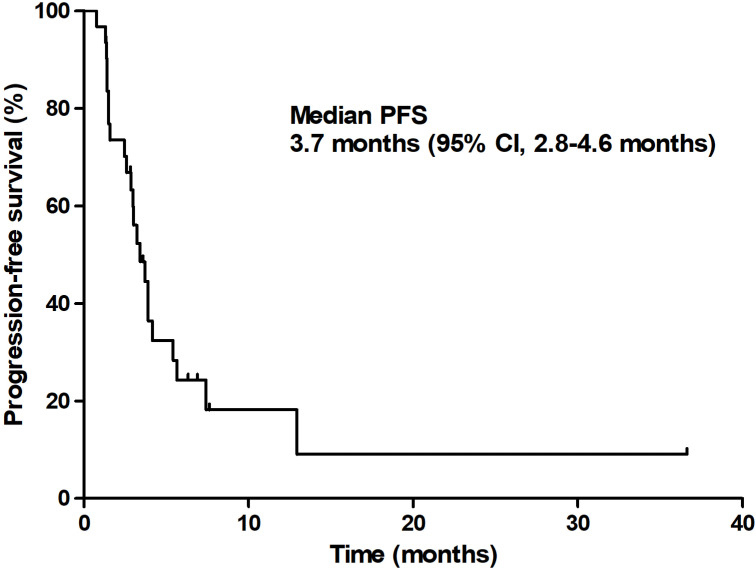
Progression-free survival.

**Figure 2:**
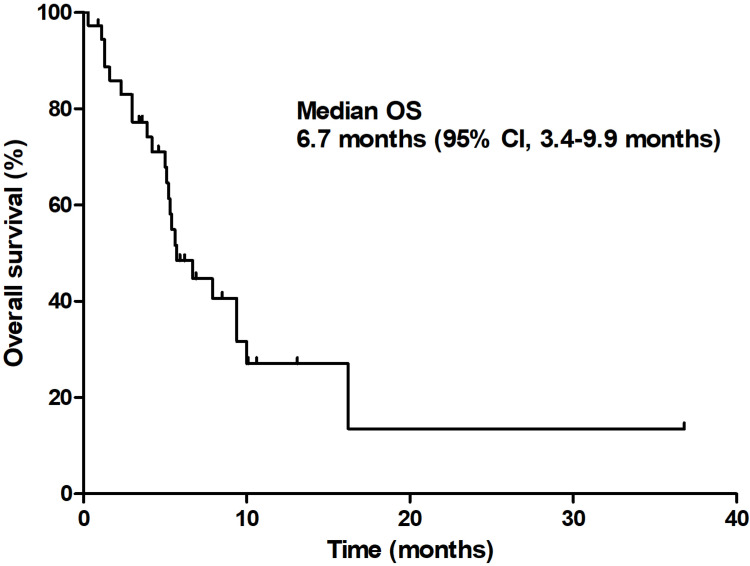
Overall survival.

At the time of analysis, 13 patients (35.1%) were still alive with a median follow-up duration of 12.9 months (95% CI 9.6-16.3 months). The median PFS was 3.7 months (95% CI 2.8-4.6 months) and the median OS was 6.7 months (95% CI 3.4-9.9 months; **Figures 1 & 2**).

### Toxicities

Safety was assessed in 36 patients on the basis of 139 cycles. One patient was lost to follow-up after receiving gemcitabine on day 1 of the first cycle, was excluded. One patient died suddenly of abdominal hemorrhage due to hepatic tumor rupture on day 3 of the first cycle. The adverse events are listed in **Table 4.** The most common grade 3/4 hematologic toxicity was neutropenia (25.0%). Febrile neutropenia developed in one patient (2.8%), who recovered without complications. Nonhematologic toxicities were usually mild and manageable. Grade 3 toxicities with a frequency of 5% or more included fatigue, infection, vomiting, and mucositis.

## Discussion

In this study, the confirmed ORR of patients was 12.5%, which is slightly better than that of gemcitabine plus erlotinib 13 and is lower than the results of phase III studies of gemcitabine plus S-1 (GS) 21. The DCR was 71.9% (95% CI, 56.8-86.3%) and the median PFS and OS were 3.7 months (95% CI, 2.8-4.6 months) and 6.7 months (95% CI, 3.4-9.9 months), respectively. The GTS regimen showed an acceptable toxicity profile in the safety analysis. Since, at this point, FOLFIRINOX and albumin-bound paclitaxel/gemcitabine are actively used as standard treatments; the implications of this result are thought to be limited.

5-FU showed a marked synergistic cytotoxic effect with gemcitabine in pancreatic cancer cells *in vitro* 22 and S-1, which has an equivalent efficacy with a continuous 5-FU infusion in solid cancer, showed promising results in several phase II studies with an ORR of 28-48% 14-18. This study aimed to improve the efficacy of the existing treatment and to investigate a novel triple-combination regimen by adding S-1, which exhibits a synergistic effect with efficacy-proven gemcitabine plus erlotinib.

While this study was in progress, the results of a phase III study (GEST study) comparing GS with gemcitabine alone were published 21. In this trial, despite the improvement in PFS and ORR, GS showed numerically longer OS compared to gemcitabine alone, but it was not statistically significant (10.1 months vs. 8.8 months, *p*=0.15). In the subgroup analysis of GEST study, GS was associated with significantly improved OS in locally advanced disease compared to metastatic disease. Furthermore, a pooled analysis of subsequent randomized studies comparing GS to gemcitabine alone also re-confirmed that GS showed better OS in locally advanced disease than metastatic disease (16.4 months vs. 11.8 months, HR 0.708, *p*=0.02) and supported the result of the subgroup analysis from the GEST study. Since most of the participants in this study had recurrent or metastatic disease rather than locally advanced disease, it is assumed that the differences in characteristics of the study population may lead to unsatisfactory results. In our study, the best response of all three patients with locally advanced disease was stable disease but it is difficult to determine statistical significance because the number of patients was too small.

Recently, it was recommended that patients with locally advanced disease should be studied separately from those with metastatic disease because locally advanced and metastatic disease are considered to be two different clinical entities, each with distinctive clinical characteristics 23. Therefore, a study design with an appropriately selected population will be required to further clarify the efficacy of the GTS regimen.

In this study, the median age of the patients was >60 years, and 75% of the patients were symptomatic at the beginning of the study. More than half of the patients had two or more metastatic sites, and 45% and 13% of patients presented with liver metastasis and peritoneal metastasis, respectively. The patients' demographics in our study are relatively inferior to the conditions of other studies, and these differences may have influenced the outcome.

Regarding the safety analysis, GTS showed a modest toxicity profile. Except for neutropenia (25%) and fatigue (22%), the incidence of all other G3 or 4 toxicity profiles did not exceed 10%, which was similar or relatively lower than that of gemcitabine plus erlotinib 13 and GS 21.

## Conclusion

In conclusion, GTS did not show the expected efficacy outcome with a confirmed ORR of 12.5%. However, considering the meaningful effect that GS showed in locally advanced disease in a subsequent study and the modest safety profile that our study showed, there may be room to further investigate the GTS regimen depending on the profile of the patient.

## Figures and Tables

**Table 1:** Patient characteristics (n=37)

Characteristics	No. of patients (%)
Age, median (range)	61.5 (35-88)
**Gender**	
Male	16 (43.2%)
Female	21 (56.8%)
**Performance status (ECOG)**	
0	10 (27.0%)
1	27 (73.0%)
**Location of primary tumor site**	
Head	9 (24.3%)
Body	7 (18.9%)
Tail	9 (24.3%)
Diffuse	4 (10.8%)
Unknown	8 (21.6%)
**Histology**	
Well differentiated	5 (13.5%)
Moderately differentiated	9 (24.3%)
Poorly differentiated	4 (10.8%)
Undifferentiated	1 (2.7%)
Unknown	18 (48.6%)
**Disease status at the time of screening**	
Locally advanced	3 (8.1%)
Metastatic	26 (70.3%)
Recurrence after curative surgery	8 (21.6%)
**Metastatic sites**	
Lymph node	16 (43.2%)
Liver	16 (43.2%)
Lung	11 (29.7%)
Peritoneum	10 (27.0%)
Others	8 (21.6%)
**No. of metastatic sites**	
1	14 (41.2%)
2	11 (32.4%)
≥3	9 (26.5%)

ECOG: Eastern Cooperative Oncology Group.

**Table 2:** Duration of drug administration and dose intensity

Criteria	
No. of cycles	140
Median cycles	3.8 (1-8)
No. of patients with dose reduction	11
Relative dose intensity for gemcitabine, Mean (range)	0.87 (0.81-0.93)
Relative dose intensity for S-1, Mean (range)	0.92 (0.87-0.96)

**Table 3:** Treatment efficacy result

Response	No. of patients
Complete response	0
Partial response	4
Stable disease	19
Progressive disease	9
Overall response rate (Confirmed)	12.5% (95% CI, 5.1-28.9%)
Disease control rate	71.9% (95% CI, 56.8-86.3%)

**Table 4:** Incidence of grade 3/4 adverse events

Grade 3/4 adverse events	Number of patients (%) (Total N=36)
**Hematologic**	
Neutropenia	9 (25%)
Febrile neutropenia	1 (2.8%)
Thrombocytopenia	1 (2.8%)
**Non-hematologic**	
Fatigue	8 (22.2%)
Infection	3 (8.3%)
Vomiting	2 (5.6%)
Mucositis	2 (5.6%)
Nausea	1 (2.8%)
Diarrhea	1 (2.8%)
Hepatopathy	1 (2.8%)
Others	4 (11.1%)
